# Analyzing and forecasting global cervical cancer burden based on WHO’s elimination strategy: insights and projections from a 1990–2021 global burden of disease (GBD) study covering 204 countries and territories

**DOI:** 10.1016/j.jare.2025.09.038

**Published:** 2025-09-25

**Authors:** Yedong Huang, Wenyu Lin, Xiaoyun Chen, Xiangqin Zheng, Huan Yi, Lin Zhang

**Affiliations:** a Fujian Maternity and Child Health Hospital College of Clinical Medicine for Obstetrics & Gynecology and Pediatrics, Fujian Medical University, Fuzhou, China; b National Key Gynecology Clinical Specialty Construction Institution of China, Fujian Provincial Key Gynecology Clinical Specialty, Fujian Maternity and Child Health Hospital, Affiliated Hospital of Fujian Medical University, Fuzhou, China; c Medical Genetic Diagnosis and Therapy Center, Fujian Maternity and Child Health Hospital, College of Clinical Medicine for Obstetrics & Gynecology and Pediatrics, Fujian Medical University, Fujian Provincial Key Laboratory of Prenatal Diagnosis and Birth Defect, Fuzhou, China; d The School of Clinical Medicine, Fujian Medical University, Department of Respiratory and Critical Care Medicine, Fujian Provincial Geriatric Hospital, Fuzhou, China; e Department of Epidemiology and Preventive Medicine, The School of Public Health and Preventive Medicine, Monash University, Australia; f Suzhou Industrial Park Monash Research Institute of Science and Technology, Monash University, China

**Keywords:** Cervical Cancer, Global Burden of Disease (GBD), World Health Organization (WHO), Epidemiology, Forecast

## Abstract

**Introduction:**

Cervical cancer remains a major global public health challenge. In 2018, the WHO launched a strategy to eliminate cervical cancer, emphasizing the need for precise epidemiological assessments.

**Objectives:**

This study utilized Global Burden of Disease (GBD) data to analyze the epidemiological indicators, geographical patterns, and temporal trends of cervical cancer, evaluating the global impact of the WHO’s strategy.

**Methods:**

Data on cervical cancer from 204 countries and territories (from 1990 to 2021) were retrieved from the GBD database. The year 2018, marking the WHO’s global call for elimination, was used to divide data into two periods: 1990 to 2018 and 2019 to 2021. Trends in age-standardized incidence rates and disability-adjusted life years (DALYs) were assessed using estimated annual percentage changes (EAPCs) and joinpoint regression. The socio-demographic index (SDI) was regressed against epidemiological indicators to explore disparities. Health inequality analyses recommended by the WHO were conducted, and an autoregressive integrated moving average (ARIMA) model was applied to predict future trends.

**Results:**

From 1990 to 2021, global age-standardized incidence rates and DALYs declined. During 1990 to 2018, the highest EAPC values were observed in Southern Sub-Saharan Africa (2·18), East Asia (0·69), and Eastern Europe (0·31). After 2019, a more significant improvement was noted globally. Regression analyses revealed an SDI-related gradient, with low-SDI countries lagging. Health inequality analyses showed widening disparities. ARIMA projections indicated stable incidence rates in most regions but a continued decline in DALYs.

**Conclusion:**

The WHO’s strategy has contributed to a notable decline in cervical cancer burden, yet challenges persist in low-SDI regions. Strengthening global cooperation and resource allocation is essential for achieving elimination goals.

## Highlights

• First global analysis of cervical cancer trends since WHO's 2018 initiative.
• Highlights regional disparities in cervical cancer burden using GBD data.
• Identifies high-potential countries for targeted cervical cancer elimination.

## Graphical abstract

**Figure:**
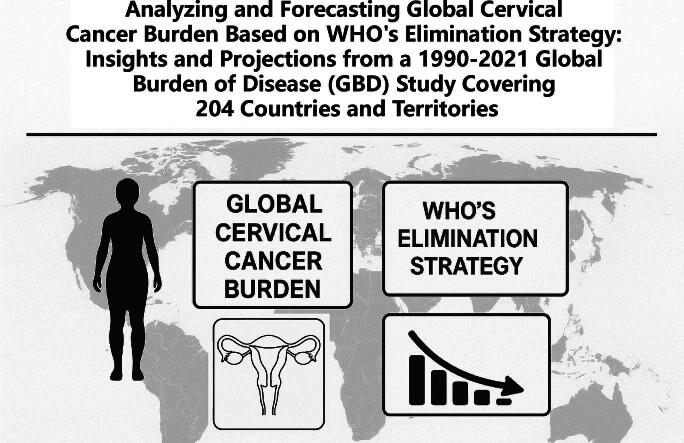

## Introduction

Cervical cancer is one of the malignancies that pose a severe threat to women's lives [1]. According to the latest GLOBOCAN (Global Burden of Cancer) data, there were approximately 661,000 new cases and 348,000 deaths from cervical cancer globally in 2022 [2]. Cervical cancer ranked fourth among all cancers in women worldwide in both incidence and mortality, accounting for 6·8% of total female cancer incidence and 8·1% of total female cancer mortality [2].

Approximately 99·7% of cervical cancer cases are caused by persistent infections with high-risk human papillomavirus (HPV) [3]. The progression from HPV infection to precancerous lesions and ultimately to cervical cancer typically spans 10–20 years [4]. As indicated by the American Cancer Society data, patients with distant − metastatic cervical cancer have a strikingly low 5 − year relative survival rate of only 19 % [5]. The unique natural history of cervical cancer provides opportunities for prevention [6]. Consequently, cervical cancer can be prevented through HPV vaccination (primary prevention) and screening (secondary prevention), and can be cured if detected early and treated appropriately [7]. However, in low- and middle-income countries (LMICs), the lack of vaccination programs, organized screening, and effective treatment options contributed to 85 %–90 % of new cervical cancer cases and deaths [8,9]. In these countries, the case fatality rate for cervical cancer exceeds 60 %, more than double that of many high-income countries [10]. Unfortunately, the global burden of cervical cancer is projected to continue rising, and without intervention, it is estimated that by 2030, the global burden will increase to nearly 700,000 cases and 400,000 deaths, representing increases of 21 % and 27 % respectively [11,12].

In light of the prolonged and substantial global burden imposed by cervical cancer, countries worldwide have committed to forging new paths towards its elimination. In 2018, Dr Tedros Adhanom Ghebreyesus, Director-General of WHO, issued a call to action for the elimination of cervical cancer [13]. In 2020, the WHO launched the official document *Global strategy to accelerate the elimination of cervical cancer as a public health problem* [14], aiming to reduce the global incidence of cervical cancer to fewer than 4 cases per 100,000 women by 2030. This strategy, known as the “90-70-90″ targets, sets a path towards the elimination of cervical cancer for all countries: 90 % of girls to be vaccinated against HPV by the age of 15, 70 % of women to be screened with a high-performance test (such as HPV DNA testing) at ages 35 and 45, and 90 % of women diagnosed with cervical lesions to receive treatment [14]. Therefore, a clear target, available technologies, and a reliable pathway for the prevention and control of cervical cancer have been established, marking a new era of cervical cancer control. To achieve this goal, there is an urgent need for an accurate assessment of the global epidemiological indicators and effectiveness of cervical cancer elimination strategy, and identifying gaps and improving measures for cervical cancer prevention and control.

Currently, the majority of epidemiological studies on cervical cancer are confined to specific countries or regions [[15], [16], [17]]. Although GLOBOCAN regularly provides estimates of global incidence and mortality rates for cervical cancer, it does not offer estimates of temporal and geographical trends or disability-adjusted life years (DALYs) [18,19]. Fortunately, the Global Burden of Disease (GBD) study collects disease data from most countries and regions around the world, enabling researchers to assess cervical cancer burden in different areas and at different times in terms of cancer incidence, mortality, years of life lost (YLLs), years lived with disability (YLDs), and DALYs [20,21]. This undoubtedly provides an excellent platform for exploring the epidemiological trends in cervical cancer.

To assess the epidemiological trends and preventive effects of cervical cancer following the introduction of the WHO strategy in 2018, this study analyzed age-standardised incidence and DALY rates across 204 countries and regions from 1990 to 2021, with a focus on temporal and geographical patterns before and after 2018. Key analytical approaches included joinpoint regression and SDI-based correlation analysis to examine disparities in disease burden [22]. This study aims to provide a clear picture of the global status of cervical cancer prevention and control in the context of the WHO strategy, and offer valuable insights for policymakers and researchers to optimize cervical cancer prevention and control strategies and reduce health inequalities worldwide.

## Material and methods

### Data sources

Data on cervical cancer covering the period from 1990 to 2021 were obtained from the GBD database, hosted by the Institute for Health Metrics and Evaluation (IHME) at healthdata.org. This comprehensive dataset covers 204 countries and territories, ensuring a broad geographical representation (**Supplementary Material 1**). The study focuses exclusively on females across all age groups, capturing the full age spectrum to provide a comprehensive understanding of cervical cancer trends. The researchers had full access to the GBD data used in this study, and the downloaded data required no further cleaning. This study did not involve linkage with other databases.

The data extracted included key epidemiological measures such as incidence, prevalence, mortality, and disability-adjusted life years (DALYs). These indicators allow for a multifaceted assessment of the burden of cervical cancer, facilitating cross-regional and temporal comparisons. To ensure data robustness, all metrics were stratified by year, region, and age group, enabling detailed trend analyses and the identification of patterns specific to different populations and time periods. The use of GBD data, which are systematically derived from multiple data sources and subjected to rigorous statistical modeling and validation, ensures the reliability and comparability of the findings [23].

### Data analysis

The age-standardised rate (ASR) was used to compare epidemiological indicators across populations with differing age structures, by employing a standard population to eliminate the impact of varying age distributions, thereby facilitating fairer and more accurate comparisons between studies. The ASR (per 100,000) was calculated by summing the product of the specific rates for each age group (a_i_, where i denotes the age group) and the corresponding number (or weight) of individuals in the same age group in the selected reference population (w_i_), and then dividing by the total sum of the standard population weights:

$$\mathrm{ASR}=\frac{\sum_{i=1}^{A}{a_{i}w}_{i}}{\sum_{i=1}^{A}w_{i}}*100,000$$

The estimated annual percentage change (EAPC) is a statistical measure used to describe the average annual rate of increase or decrease over a specified period for a given indicator, such as incidence, mortality, or survival rates of a disease. This metric is commonly used in epidemiological research to quantify the temporal trends of health indicators. The formula for calculating the EAPC is as follows (where β represents the slope in the regression model):

$$\mathrm{EAPC}=(e^{\beta }-1)*100$$

Following the WHO’s call in May 2018 for the global elimination of cervical cancer, this study compares the periods from 1990 to 2018 and from 2019 to 2021, and maps the epidemiology of cervical cancer worldwide to examine the impact of this strategy on a global scale. Additionally, the study employs joinpoint regression models to monitor and analyse trends in the incidence and DALYs of cervical cancer globally, aiding researchers in understanding changes in data trends and thus providing an objective assessment of the impact of the WHO’s elimination strategy.

Considering the economic disparities among countries and regions worldwide, this study utilised the Socio-Demographic Index (SDI) to quantify the development status of the countries and regions included in the research and conducted regression and frontier analysis to correlate these with incidence rates and DALYs. The SDI is a composite measure used to assess a country or region’s performance across multiple domains of social development, including education, economic status, and fertility levels [24,25]. Countries or regions are classified into five tiers based on their SDI values: Low SDI, Low-middle SDI, Middle SDI, High-middle SDI and High SDI (according to the GBD official documentation, the cutoff values for these five tiers are: 0, 0·4658, 0·6188, 0·7120, 0·8103 and 1, respectively). Data on the SDI for each country or region can be retrieved or downloaded from the relevant websites (Global Burden of Disease Study 2021 (GBD 2021) Socio-Demographic Index (SDI) 1950–2021 | GHDx (healthdata.org)).

In frontier analysis, the observed incidence or DALYs of a country or region, compared to its corresponding frontier, are defined as the effective difference, representing the health gains yet to be realised based on the current level of development of that country or region. Therefore, this method was used to assess the performance of certain countries or regions in cervical cancer prevention and control under their present SDI conditions.

Additionally, in accordance with WHO recommendations, this study used the Slope Index of Inequality (SII) and the Concentration Index (CI) to assess absolute and relative income-related inequalities between countries and regions (Handbook on health inequality monitoring with a special focus on low- and middle-income countries). To investigate and quantify disparities in the epidemiological indicators of cervical cancer across countries or regions with varying SDI levels, this study conducted an analysis of health inequalities in incidence and DALYs for 204 countries or regions, in accordance with WHO recommendations.

The Autoregressive Integrated Moving Average Model (ARIMA) is a statistical model widely employed for the analysis and forecasting of time series data, and has frequently been used in studies related to the Global Burden of Disease (GBD) [26,27]. This study forecasted the epidemiological trends of cervical cancer over the next 30 years based on global data from 1990 to 2021, segmented by various levels of SDI to enhance understanding and evaluation of how different levels of social development might influence these trends in the coming decades.

Data were analysed using R software (version 4·1·2) and the joinpoint software (version 5·2·0), which was downloaded from the official website (Joinpoint Regression Program (cancer.gov)). The R packages used included the following: data.table, dplyr, Epi, ggplot2, ggsci, magrittr, RColorBrewer, readxl, factoextra, openxlsx, purr, tidyr, ggpubr, ggrepel, parallel, broom, car, MASS, mgcv, splines, ggmap, tidyverse, maps, rgdal, reshape, forecast, and splines.

## Results

### Global, regional and national cervical cancer burden across different time periods

Tables 1 **and S1** present age-standardised incidence rates and disability-adjusted life years (DALYs) for cervical cancer across 21 global regions and worldwide, for the periods from 1990 to 2018 and from 2019 to 2021. Overall, both incidence and DALYs showed a general decline during these periods. Between 1990 and 2018, Southern Sub-Saharan Africa, East Asia, and Eastern Europe had the highest EAPC in age-standardised incidence rates, with values of 2·18 (95 % CI: 1·66-2·7), 0·69 (95 % CI: 0·51-0·88), and 0·31 (95 % CI: 0·17-0·44), respectively (Table 1). Most regions showed a decline in age-standardised incidence, as indicated by negative EAPC during this period (Table 1). From 2019 to 2021, the decline accelerated in most regions, with increasingly negative EAPCs (Table 1). The regions with the highest EAPC in global age-standardised incidence were Tropical Latin America (1·81 [95 % CI: 1·3-2·31]), Southern Sub-Saharan Africa (0·44 [95 % CI: −0·34-1·23]), and Central Sub-Saharan Africa (0·35 [95 % CI: 0·09-0·6]) (Table 1).

**Table 1:** Age-standardized rates (per 100,000), absolute number of incidence of cervical cancer of 1990–2018 and 2019–2021, and their estimated annual percentage changes (EAPC).

Location	1990–2018					2019–2021				
	Num_1990_CI	ASR_1990(per 100,000)_CI	Num_2018_CI	ASR_2018(per 100,000)_CI	EAPC_CI	Num_2019_CI	ASR_2019(per 100,000)_CI	Num_2021_CI	ASR_2021(per 100,000)_CI	EAPC_CI
Global	409548·5 (383207·2-438505·6)	18·1 (16·9-19·4)	642426·4 (596389·2-687439)	15·5 (14·4-16·6)	−0·58 (−0·68–0·49)	651592·1 (600690·6-701806·1)	15·5 (14·3-16·6)	667426·4 (613030·1-726422·1)	15·3 (14·1-16·7)	−0·45 (−0·95-0·05)
Andean Latin America	4153·9 (3640·4-4664·9)	33·2 (29·1-37·3)	9208·5 (7743·8-10757·5)	30·5 (25·6-35·6)	−0·75 (−0·92–0·59)	9988·9 (8090·5-12034·7)	32·1 (26-38·6)	9757·2 (7475·6-12359)	29·8 (22·8-37·7)	−3·48 (−6·25–0·62)
Australasia	2001·2 (1863·3-2175·2)	17·2 (16-18·7)	1725·8 (1564·6-1885·4)	8·9 (8·1-9·8)	−1·82 (−2·1–1·53)	1782·2 (1607·4-1948·8)	9·1 (8·2-9·9)	1751·6 (1575·8-1924·7)	8·5 (7·7-9·4)	−2·75 (−4·9–0·55)
Caribbean	4635·3 (4190·6-5197·1)	31·5 (28·5-35·2)	7321·4 (6386·9-8315·6)	28·6 (24·8-32·5)	−0·46 (−0·55–0·38)	7484·9 (6462·2-8638·1)	28·8 (24·8-33·2)	7381·7 (6192·2-8712·1)	27·6 (23·1-32·6)	−2·01 (−3·36–0·64)
Central Asia	5168·9 (4951·5-5420·2)	18·2 (17·4-19·1)	7191·6 (6760·6-7629·5)	15·1 (14·2-16)	−0·34 (−0·52–0·16)	7190·4 (6678·9-7739)	14·8 (13·7-15·9)	7178·8 (6265·6-8081·5)	14·2 (12·4-15·9)	−1·99 (−3·02–0·95)
Central Europe	16319·8 (15590·6-17021·3)	21·7 (20·7-22·6)	15271·1 (14454·3-16028·4)	17·4 (16·5-18·2)	−0·86 (−1·06–0·66)	14,755 (13874·3-15538·9)	16·7 (15·8-17·6)	14,203 (12928·3-15545·2)	15·9 (14·5-17·5)	−2·27 (−5·49-1·07)
Central Latin America	22434·4 (21858·4-23000·3)	41·9 (40·6-42·8)	37740·1 (35893·5-39660·1)	28·7 (27·3-30·2)	−1·68 (−1·86–1·5)	38,656 (36578·7-41397·2)	28·8 (27·2-30·8)	40,343 (34553·6-46252)	28·9 (24·8-33·1)	0·18 (−0·39-0·76)
Central Sub-Saharan Africa	6099·6 (4558·3-7964·2)	39·4 (29·6-51·3)	13728·5 (9530·6-18473·5)	37·8 (26·3-50·2)	−0·18 (−0·21–0·14)	14218·7 (9824·9-18992·5)	37·7 (26·2-50·3)	15328·2 (10593·8-20548·6)	38 (26·3-51)	0·35 (0·09-0·6)
East Asia	61909·1 (50407·5-76000·8)	12·2 (9·9-14·9)	135649·1 (102439·8-172586·4)	13·7 (10·3-17·4)	0·69 (0·51-0·88)	136929·6 (104201·3-172568)	13·6 (10·3-17·2)	137863·8 (101144·5-177754·7)	13·4 (9·9-17·4)	−0·84 (−0·84–0·83)
Eastern Europe	23498·9 (22662·3-24258·8)	14·9 (14·4-15·4)	25136·2 (24230·3-26133·9)	16·4 (15·9-17)	0·31 (0·17-0·44)	25393·6 (24428·1-26586·7)	16·6 (16-17·3)	25339·2 (22882·9-27756)	16·5 (14·8-18·1)	−0·28 (−2·29-1·77)
Eastern Sub-Saharan Africa	22644·1 (18815·5-27483)	45·7 (37·9-55·5)	37279·7 (30503·3-48234·4)	33·5 (27·6-42·9)	−1·41 (−1·53–1·28)	38597·4 (31149·7-49518·4)	33·5 (27·2-42·4)	41,370 (33124-52883·5)	33·4 (27·2-42·1)	−0·01 (−0·01–0·01)
High-income Asia Pacific	12720·4 (12005·8-13515·6)	11·9 (11·2-12·6)	15733·3 (14075-16912·1)	11·1 (10·2-11·8)	0·02 (−0·1-0·14)	15900·5 (14295·4-17148·4)	11·3 (10·4-12·1)	15577·8 (14044·1-16881·5)	11 (10·3-11·9)	−1 (−1·05–0·95)
High-income North America	32695·4 (31769·2-33479·7)	19·6 (19·1-20·1)	30768·1 (29697·3-31638·8)	13·3 (12·9-13·6)	−1·34 (−1·57–1·1)	30619·3 (29541·7-31469·1)	13·1 (12·7-13·4)	30415·2 (29096·1-31621·8)	12·7 (12·2-13·2)	−1·34 (−1·42–1·26)
North Africa and Middle East	6235·2 (5462·1-7543·8)	6 (5·2-7·2)	12159·2 (10569·8-13768·7)	4·8 (4·2-5·5)	−0·62 (−0·67–0·58)	12473·6 (10831·5-14242·9)	4·8 (4·2-5·5)	12912·9 (11008·4-15181·6)	4·7 (4-5·5)	−0·9 (−1·32–-0·47)
Oceania	668·3 (506·1-928)	34·2 (26·8-47·6)	1252·9 (978·1-1809·7)	27·4 (21·5-39·2)	−0·84 (−0·9–0·78)	1313 (1025·4-1905·3)	27·7 (21·9-40·4)	1372·2 (1070·2-2017·1)	27·3 (21·5-39·7)	−0·75 (−0·81–0·69)
South Asia	83506·8 (69576·8-96968·9)	23·7 (19·5-27·5)	125739·1 (112115·9-139565·2)	16 (14·3-17·7)	−1·64 (−1·97–1·32)	126688·9 (112972·8-142161·7)	15·7 (14-17·7)	132,482 (114540·7-151294·7)	15·5 (13·5-17·7)	−0·52 (−0·55–0·49)
Southeast Asia	30364·6 (26327·2-34797·6)	18·1 (15·6-20·6)	54562·7 (47675·1-61772·1)	15·3 (13·4-17·3)	−0·74 (−0·86–0·62)	55973·8 (48224·9-64821·9)	15·3 (13·2-17·7)	58017·1 (49190·9-67747·1)	15·2 (12·9-17·7)	−0·4 (−0·76–0·04)
Southern Latin America	6030·1 (5640·9-6407·3)	24·4 (22·8-26)	9744·2 (9033·2-10458·7)	25 (23·2-26·9)	−0·22 (−0·4–0·04)	9886·3 (9121·9-10596·3)	25 (23·1-26·8)	9302·1 (8566·1-10068·3)	22·8 (21·1-24·7)	−4·31 (−5·31–3·31)
Southern Sub-Saharan Africa	5528·2 (4617·7-6832·4)	29·9 (25·1-37·3)	15071·3 (13424·8-16619·6)	42·6 (38·1-46·6)	2·18 (1·66-2·7)	15369·8 (13791·2-17098·4)	42 (37·8-46·6)	16246·7 (14189-18417·6)	42·4 (37·2-47·9)	0·44 (−0·34-1·23)
Tropical Latin America	13345·9 (12840·5-13859·2)	23·1 (22·1-24)	25083·7 (24015·9-25992·9)	19·3 (18·5-20)	−0·96 (−1·11–0·8)	25857·3 (24845·1-26837·3)	19·5 (18·8-20·3)	27,823 (26275·2-29199·3)	20·3 (19·2-21·3)	1·81 (1·3-2·31)
Western Europe	35616·4 (34385·6-36706)	14·2 (13·8-14·7)	30188·3 (28261·2-31482·7)	9·6 (9·1-9·9)	−1·23 (−1·46–1)	29762·9 (27797-31205·8)	9·4 (9-9·8)	27921·5 (26003·1-29390·6)	8·7 (8·3-9·1)	−3·47 (−7·07-0·27)
Western Sub-Saharan Africa	13972·2 (11424·7-17090·4)	26·2 (21·4-31·7)	31871·8 (25131·6-39292·6)	24·6 (19·7-29·8)	−0·19 (−0·22–-0·16)	32750·1 (25383·7-41402·4)	24·4 (19·3-30·2)	34839·4 (26668·2-42612·9)	24·1 (18·9-29·1)	−0·49 (−0·75–0·22)

Abbreviation: ASR: Age-Standardized Rates; EAPC: Estimated Annual Percentage Changes; CI: Confidence Interval (95 %).

For age-standardised DALYs, all regions except Southern Sub-Saharan Africa (1990–2018, 2·14 [95 % CI: 1·59-2·7]) and Tropical Latin America (2019–2021, 1·14 [95 % CI: 0·74-1·56]) showed a declining trend, as reflected by negative EAPC (**Table S1**). To illustrate regional disparities, age-standardised incidence rates and DALYs are presented globally and across 21 regions in bar charts (**Fig. S1**).

**Fig. 1:**
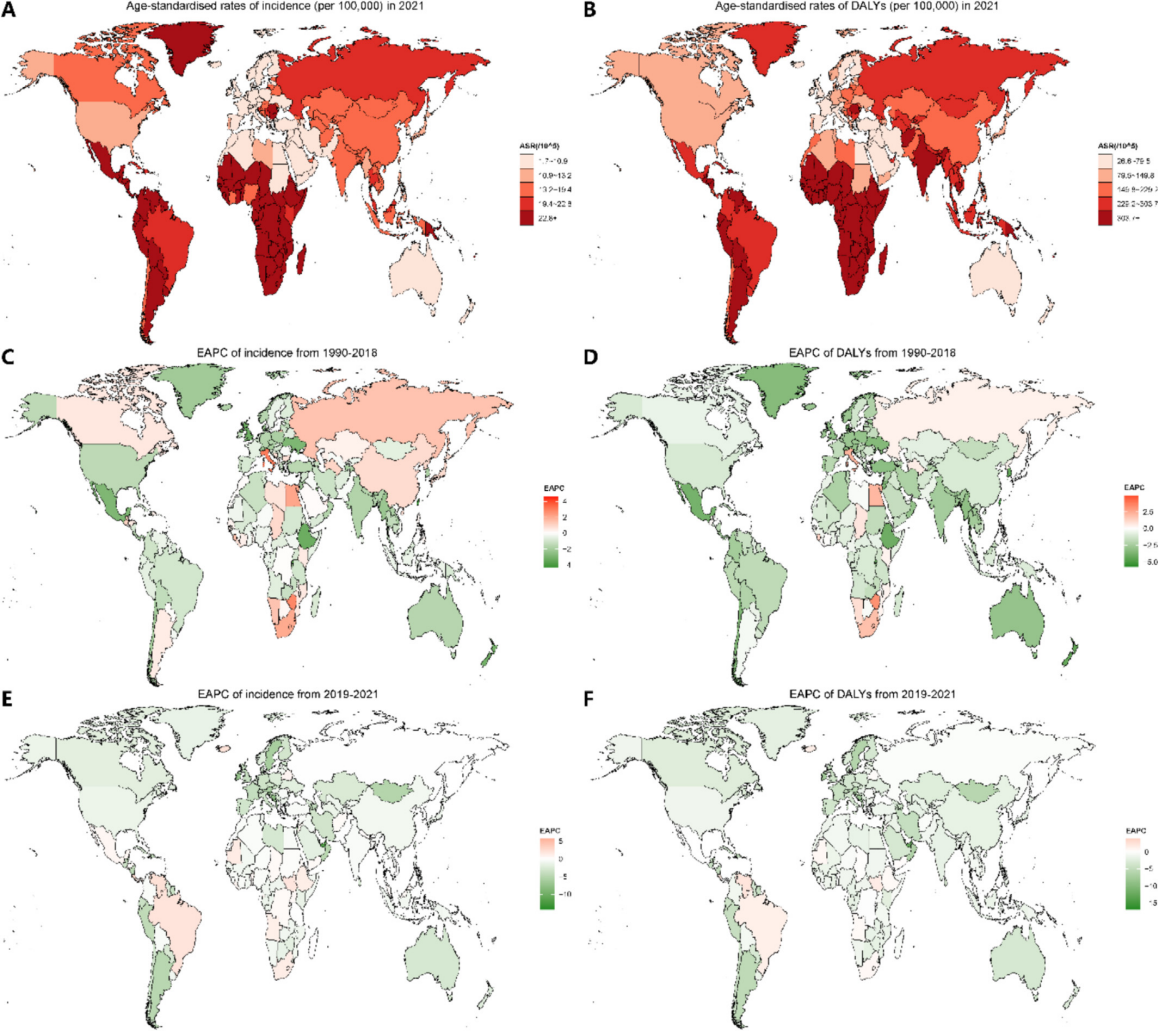
National age-standardised rates of incidence and DALYs in 2021, and their EAPC of 1990–2018 and 2019–2021 for cervical cancer. Age-standardised rates of incidence (per 100,000) **(A)** and DALYs (per 100,000) **(B)** for cervical cancer in 2021, along with the corresponding EAPC of incidence **(C)** and DALYs **(D)** from 1990 to 2018, and EAPC of incidence **(E)** and DALYs **(F)** from 2019 to 2021.

Fig. 1 shows global epidemiological maps of age-standardised incidence rates and DALYs across different time periods. Fig. 1, Fig. 1 depict age-standardised incidence and DALYs for 204 countries in 2021, revealing generally similar patterns, with higher rates observed in Arctic regions, South America, South Africa, and parts of Central Asia, South Asia, and Southeast Asia. Between 1990 and 2018, countries including Russia, Canada, China, Kazakhstan, and some in Southern Africa exhibited rising annual incidence rates (Fig. 1C). However, despite this, DALYs declined year-on-year due to improvements in medical care, with the exception of Russia, where the disease burden continued to rise (Fig. 1D). From 2019 to 2021, most countries showed negative EAPCs in both incidence and DALYs (Fig. 1, Fig. 1), indicating a global trend towards declining age-standardised rates of incidence and DALYs for cervical cancer.

## Global trends in cervical cancer burden from 1990 to 2021

**Fig. 2:**
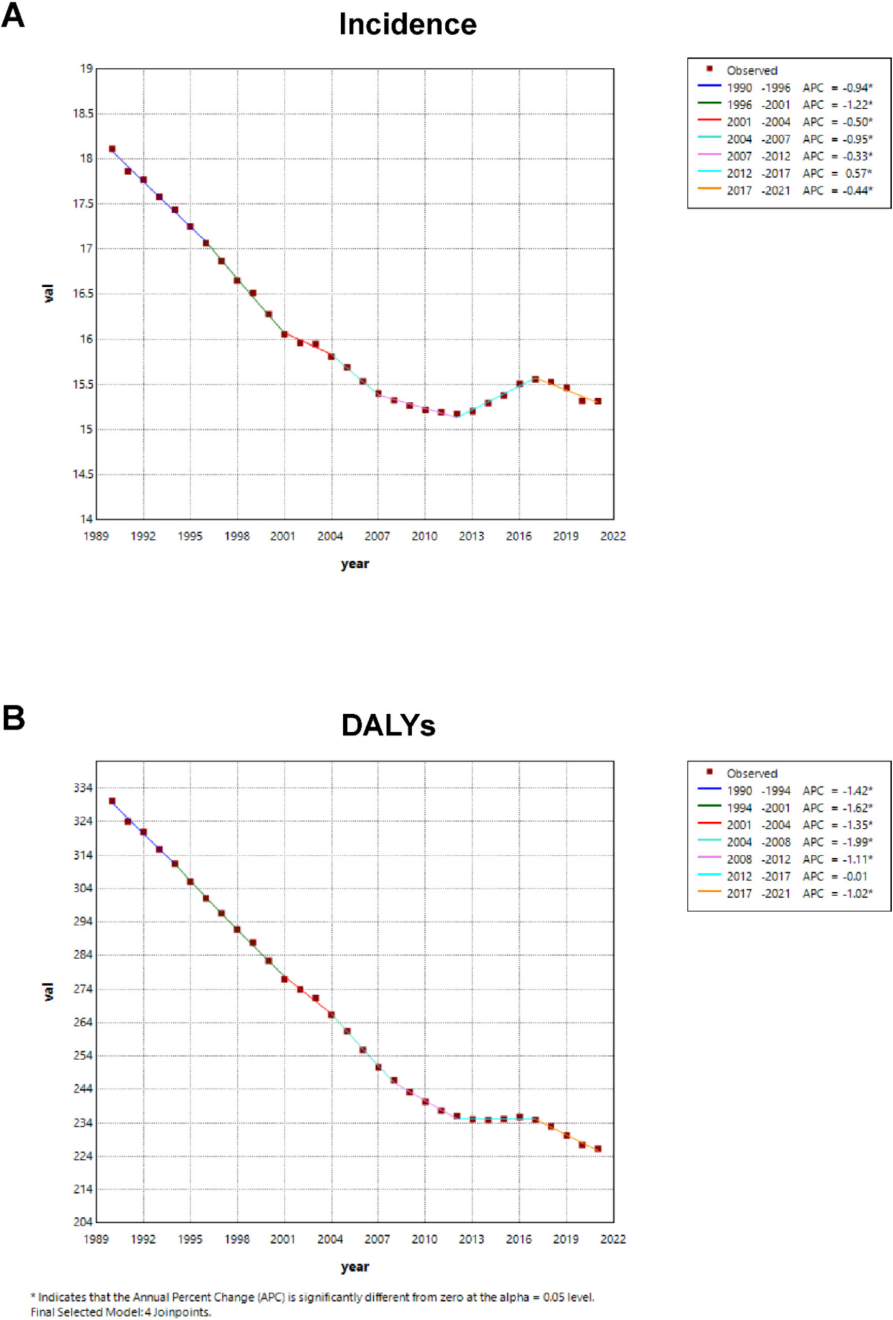
Joinpoint model of age-standardised rates of incidence (per 100,000) and DALYs (per 100,000) for cervical cancer globally from 1990 to 2021. Global age-standardised incidence rates (per 100,000) **(A)** and DALYs (per 100,000) **(B)** for cervical cancer from 1990 to 2021. Abbreviation: APC, Annual Percent Change. *: P < 0·05.

Fig. 2 displays the joinpoint model of age-standardised rates of incidence and DALYs for cervical cancer globally from 1990 to 2021. Overall, both incidence rates and DALYs showed a significant declining trend worldwide. However, during the period 2012–2017, there was a notable rebound in incidence rates (Fig. 2A), coinciding with a significant plateau in the reduction of DALYs (Fig. 2B). Notably, from 2018 onwards, both the age-standardised rates of incidence and DALYs for cervical cancer resumed their downward trajectory (Fig. 2A **and** Fig. 2B). Data from the joinpoint model of age-standardised rates of incidence and DALYs from 1990 to 2021 are uploaded as **Supplementary Material 2**.

## Cervical cancer and socio-demographic index: correlation and health potential assessment

**Fig. 3:**
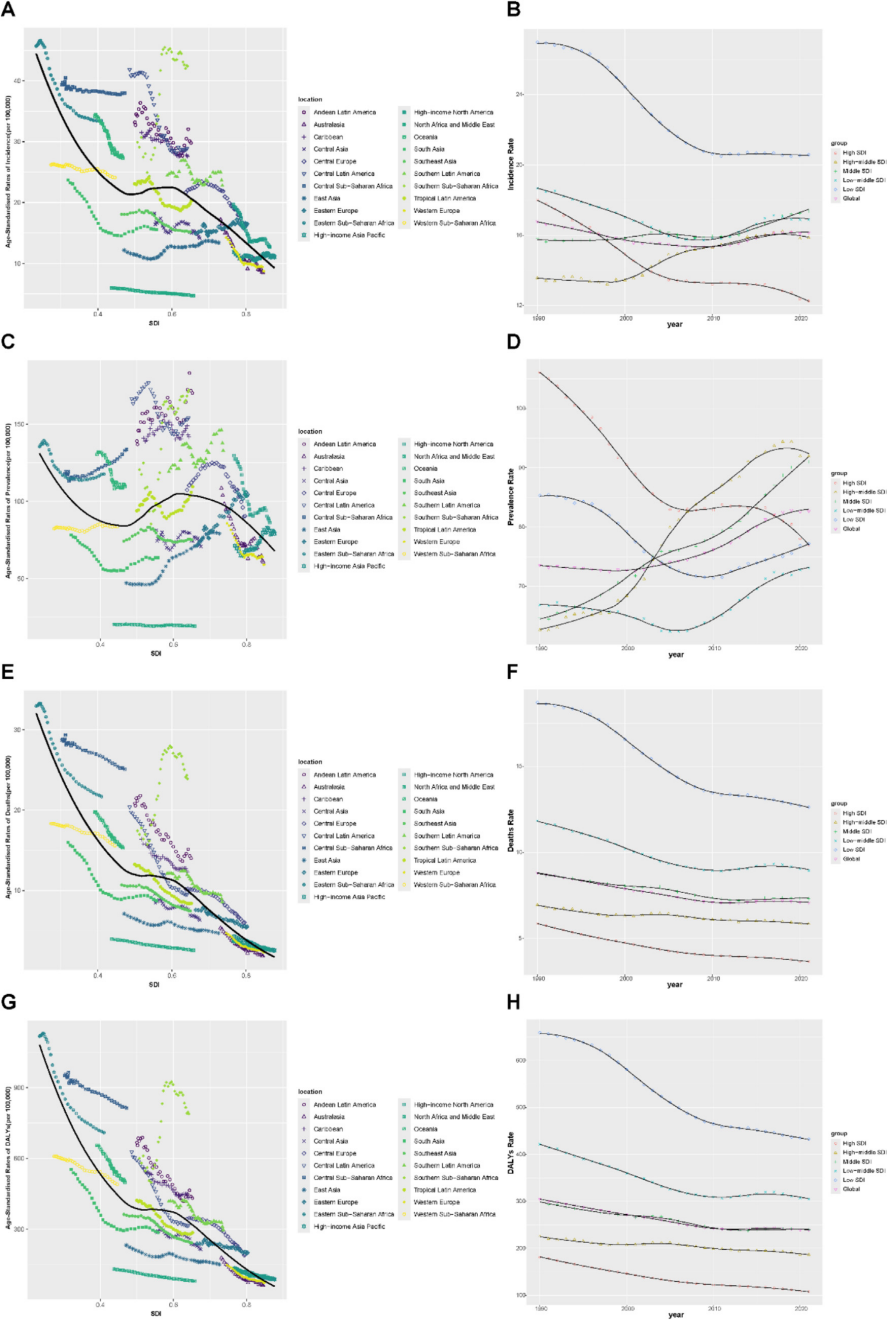
Cervical cancer burden across global regions and SDI strata. The association between SDI and four key indicators across 21 global regions and temporal trends from 1990 to 2021 (**A-B.** incidence, **C-D.** prevalence, **E-F.** deaths and **G-H.** DALYs, respectively).

A negative association was observed between SDI and four key indicators of cervical cancer burden (incidence, prevalence, deaths and DALYs, respectively) across the 21 GBD regions (Fig. 3). Regions with lower SDI values consistently showed higher levels of cervical cancer burden, with Southern Sub-Saharan Africa and Central Latin America showing the greatest clustering at the upper end of each metric. Temporal trends from 1990 to 2021 revealed pronounced inequalities across SDI strata (Fig. 3). High-SDI regions experienced early and sustained reductions in deaths and DALYs, while low-SDI and low-middle SDI regions remained at higher levels, indicating a greater disease burden. Notably, although age-standardised DALYs declined across all SDI groups, the disparities in disease burden remained substantial. The corresponding SDI data for each region from 1990 to 2021 have been uploaded as **Supplementary Material 3**.

**Fig. 4:**
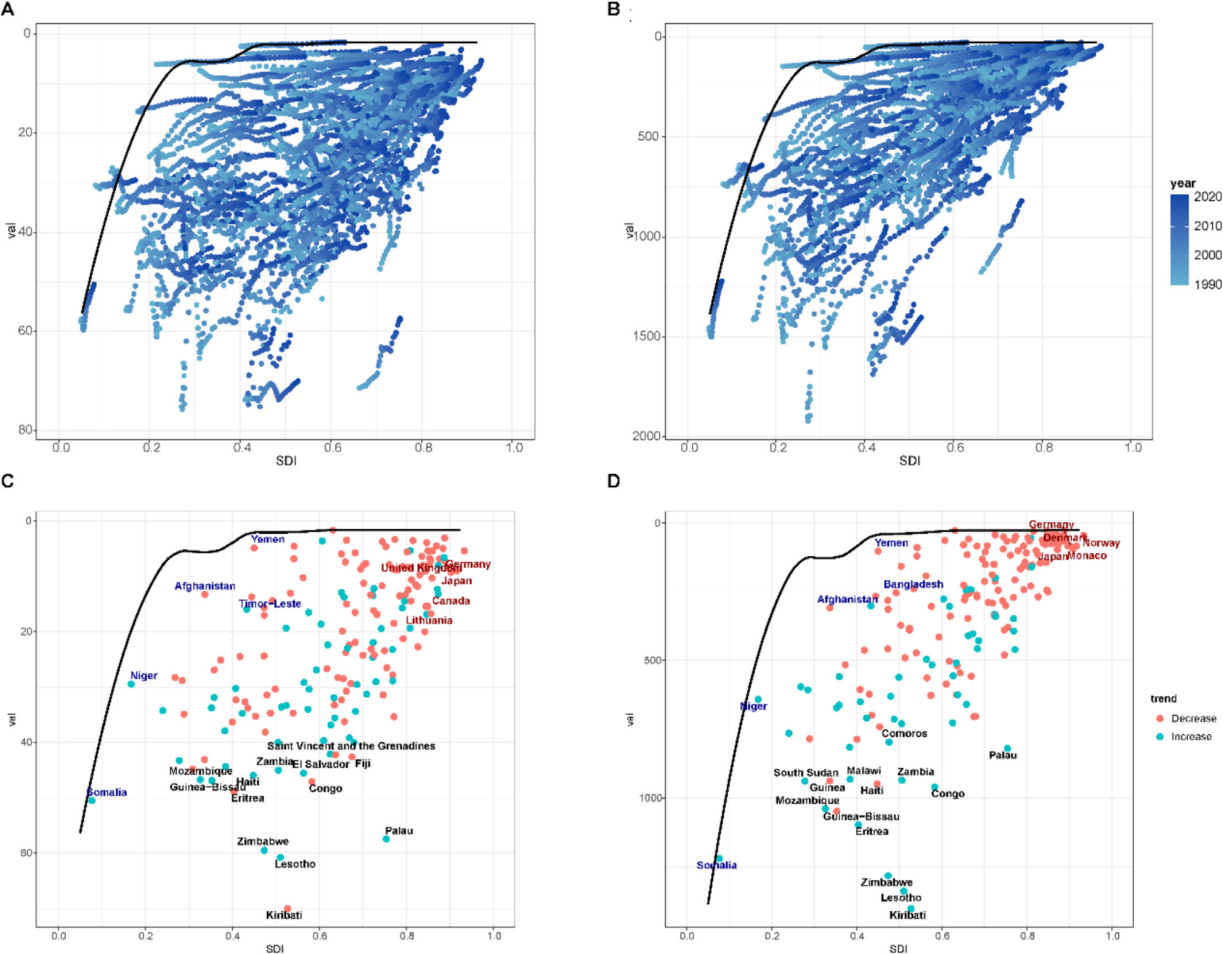
Frontier analysis based on SDI and age-standardised rates of incidence and DALYs from 1990 to 2021. Trajectory plot from 1990 to 2021 for incidence rates **(A)** and DALYs **(B)**. Scatter plot for incidence rates **(C)** and DALYs **(D)** in 2021. *Footnotes: The frontier is delineated in solid black color; countries and territories are represented as dots. The countries and territories with a relatively large effective gap (larger incidence or DALYs gap compared to the frontier than other countries) are labeled in black on the map; examples of frontier countries with low SDI (<0·5) and low effective difference are labeled in blue, and examples of countries and territories with high SDI (>0·85) and relatively high effective difference for their level of development are labeled in red.* (For interpretation of the references to color in this figure legend, the reader is referred to the web version of this article.)

In light of the disparities in development between countries and regions, this study used frontier analysis on the age-standardised rates of incidence and DALYs across 204 countries and territories globally to assess the health potential in the prevention and treatment of cervical cancer. As illustrated in Fig. 4, when the SDI reaches 0·5 or higher, the theoretical values of age-standardised incidence rates and DALYs tend to stabilise, and the impact of SDI becomes insignificant. However, in reality, most countries and regions do not achieve these theoretical values due to various constraints, leading to what is referred to as the 'Effective Difference' between actual performance and theoretical potential. Interestingly, some countries, such as Palau, have a relatively high SDI (>0·712, classified as a high-middle SDI country), yet exhibit considerable health potential in cervical cancer prevention and control (Fig. 4, Fig. 4). This may be due to the relatively limited resources allocated to cervical cancer control within these countries. In this study, countries or regions with substantial effective differences are highlighted (Fig. 4, Fig. 4). Notably, countries such as Palau, Zambia, Congo, Haiti, Guinea-Bissau, Mozambique, Eritrea, Zimbabwe, Lesotho, and Kiribati demonstrate significant health potential in terms of age-standardised rates of incidence and DALYs.

## Health inequalities in cervical cancer from 1990 to 2021

To investigate and quantify the disparities in cervical cancer incidence and disease burden across countries and regions with different SDI levels, this study used the method of health inequity analysis as recommended by the World Health Organization. The Slope Index of Inequality (SII) reflects absolute health inequality, with larger values indicating greater disparities across SDI levels. The Concentration Index (CI) measures relative inequality, ranging from −1 to 1, where positive values suggest better outcomes in higher-SDI regions and values closer to zero indicate more equity.

**Fig. 5:**
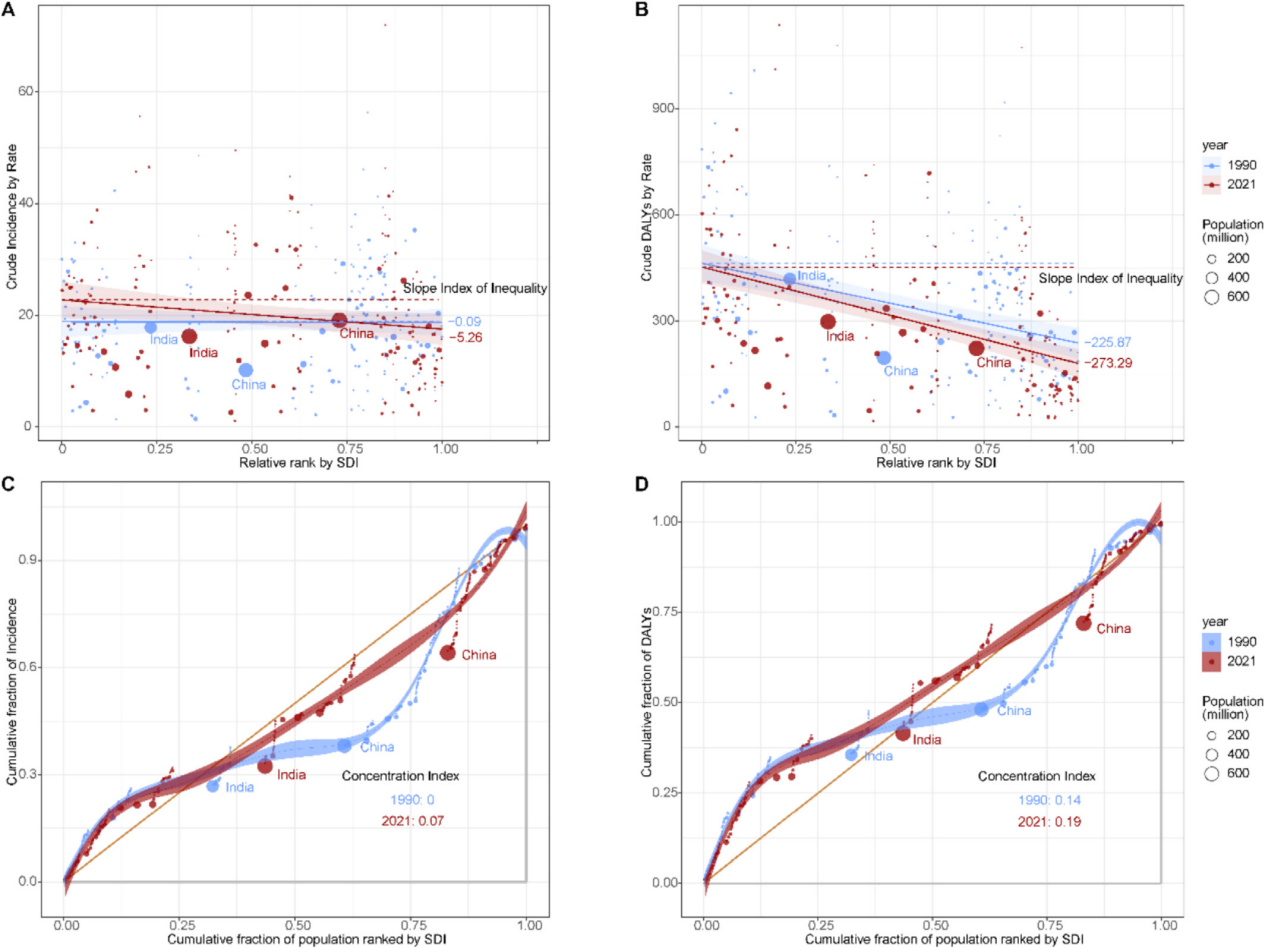
Analysis of Health Inequities in Global Cervical Cancer Incidence and DALYs per 100,000 (1990–2021). Relationship between the incidence rates **(A)** or DALYs **(B)** of cervical cancer in 1990 and 2021 and the relative ranking of SDI (the slope represents the SII value). Inequality in the incidence rates **(C)** and DALYs **(D)** of cervical cancer in 1990 and 2021 across the SDI distribution. *Footnotes: The size of the dots represents the population size of different countries. Countries with populations greater than one billion are marked on the map (China, India).*

The results indicate that both incidence rates and DALYs have shown a consistent downward trend in the SII from 1990 to 2021 (**Fig. S2 and S3**). In terms of incidence rates and DALYs, countries or regions with a high SDI undeniably have lower rates, and this inequality is intensifying (Fig. 5, Fig. 5). Regarding the CI, the results shown in Fig. 5, Fig. 5 suggest that health inequalities have worsened from 1990 to 2021, with health outcomes increasingly favoring countries or regions with higher SDI. As shown in **Table S2**, the annual variations in the SII for global cervical cancer burden indicate that, despite the implementation of the WHO strategy, the SII values for both incidence and DALYs continued to decline from 2019 to 2021, reflecting an ongoing escalation in health inequities. The specific annual data for SII corresponding to incidence rates and DALYs have been uploaded as **Supplementary Material 4**.

## Global projections of cervical cancer incidence and DALYs through 2050

This study used the ARIMA model to forecast epidemiological changes over the next 30 years based on global cervical cancer data from 1990 to 2021 (**Fig. S4**). As depicted in **Fig. S4A and S4G**, the age-standardised rates of incidence and DALYs of global cervical cancer are predicted to remain relatively stable and generally decline, respectively, over the next 30 years. In terms of age-standardised incidence rates, apart from a notable decline in high SDI regions (**Fig. S4B**), other regions are expected to maintain relative stability or experience a slight decrease over the next 30 years (**Figs. S4C-S4F**). For age-standardised DALYs, while a slight decrease is projected in low SDI regions (**Fig. S4L**), a more significant downward trend is anticipated in other regions (**Figs. S4H-S4K**). The specific values for age-standardised incidence rates and DALYs forecasted by the ARIMA model have been uploaded as **Supplementary Materials 5 and 6**.

Indeed, the GBD study has updated its official forecasting tool (https://vizhub.healthdata.org/gbd-foresight/); however, regrettably, this tool does not provide projections for incidence rates. Consequently, this study has uploaded the official forecasts for global and SDI-specific cervical cancer DALYs up to the year 2050, as well as the corresponding trend charts, as **Supplementary Material 7 and Fig. S5**.

## Discussion

This study provides an assessment of the initial effects of the “Strategy to Eliminate Cervical Cancer” (proposed in 2018) [13] by presenting the latest trends in the burden of cervical cancer across 204 countries and 21 regions worldwide following the strategy’s implementation. From 2019 to 2021, age-standardised incidence rates of cervical cancer continued to rise in Tropical Latin America and Southern Sub-Saharan Africa. In Tropical Latin America, age-standardised DALYs also increased, while other regions experienced more pronounced declines in both age-standardised incidence rates and DALYs of cervical cancer. Moreover, there is a clear socioeconomic gradient in the incidence and burden of cervical cancer; as the SDI increases, both the incidence rates and DALYs decrease, and this regional health inequality is intensifying. Less developed regions and nations such as Palau, Zambia, Congo, Haiti, Guinea-Bissau, Mozambique, Eritrea, Zimbabwe, Lesotho, and Kiribati are still far from achieving the elimination of cervical cancer targets, and there remains substantial potential for health improvements in prevention and control. Predictions from the ARIMA model indicate that the global age-standardised incidence rates of cervical cancer will remain relatively stable over the next 30 years, while age-standardised DALYs are expected to decrease.

Since 1990, global age-standardised incidence and DALY rates for cervical cancer have declined overall, reflecting worldwide prevention efforts. Notably, between 2012 and 2017, there was a rebound in the age-standardised incidence rates of cervical cancer, and a significant plateau was observed in the reduction of DALYs. Since 2018, both age-standardised incidence rates and DALYs have returned to their declining trend. This positive shift can be attributed to two key factors: the early HPV vaccination programs [28], and the WHO global strategy of cervical cancer elimination [29].

Although some countries had initiated cervical cancer prevention efforts earlier, the WHO’s elimination strategy in 2018 marked the first globally coordinated initiative with clearly defined targets and timelines. It is important to emphasize that HPV vaccination is not only a core intervention within the WHO strategy but also an inseparable component—serving as the foundational pillar of the “90–70-90” elimination framework. Accordingly, the rollout of HPV vaccination programs in high-SDI countries beginning in 2006—exemplified by the approval of the quadrivalent HPV vaccine (Gardasil) in the United States and Canada that year [30] —represented a new phase in cervical cancer prevention and laid the groundwork for the formulation and global implementation of the WHO strategy in 2018, as part of a continuous policy trajectory [31]. Therefore, when assessing the early impact of the WHO strategy, it is necessary to acknowledge that the observed decline in cervical cancer incidence from 2019 to 2021 may partly reflect the delayed effects of vaccination campaigns launched over a decade earlier in high-SDI countries. Nevertheless, 2018 remains a significant policy milestone, marking the formal launch of the WHO global elimination initiative with coordinated international efforts and defined programmatic goals. Critically, our joinpoint regression analysis statistically identified 2018 as a significant inflection point in cervical cancer burden trends, further validating the choice of this year as a demarcation in our study design. Based on this segmentation, we observed a renewed decline in both ASR of incidence and DALYs after 2018. Other studies have similarly reported that, since 2018, global implementation of cervical cancer screening and early intervention for precancerous lesions has expanded, contributing to further reductions in disease burden [32,33].

Specifically, the WHO has promoted reduction of the burden of cervical cancer through comprehensive prevention, screening, and treatment strategies. Since the launch of the global strategy, an additional 30 countries have introduced the HPV vaccine, bringing the total to 140 countries that have incorporated it into their national immunisation programmes [7,34]. Due to a global shortage of HPV vaccines, only 15 % of target-age girls were covered in 2019 [35]. The WHO proposed alternative strategies, such as targeting girls aged 13 or 14, or administering the first dose at ages 9 or 10 with a two-dose regimen spaced 3–5 years apart [36]. Increased HPV vaccine coverage has significantly reduced cervical cancer incidence [10]. The WHO strategy also emphasises screening and treatment effectiveness. In 2019, the WHO recommended thermocoagulation as an ablative treatment technique for the “screen-and-treat” or “screen-triage-treat” strategy, which enables direct treatment of women who test positive in screening [37]. Following treatment for CIN II or CIN III+, the risk of developing CIN III+ within five years is 0·9% [38]. In 2024, the WHO released preferred product characteristics for therapeutic HPV vaccines, complementing current treatment measures [39]. Collectively, WHO’s screening and treatment interventions help prevent persistent HPV infection, and further reduce the incidence of invasive cervical cancer.

From 1990 to 2018, Southern Sub-Saharan Africa, East Asia, and Eastern Europe had the highest EAPC in age-standardised incidence rates of cervical cancer (Table 1). From 2019 to 2021, Tropical Latin America, Southern Sub-Saharan Africa, and Central Sub-Saharan Africa had the highest EAPCs (Table 1), indicating effective prevention efforts in East Asia. In China, HPV vaccine doses increased from 3.417 million (2018) to 12.279 million (2020) [40], with two domestic vaccines introduced and free/subsidized programs in over 30 cities [41]. Japan’s HPV vaccination program began in 2010, but coverage declined from 70 % to less than 2 % by 2022 [42]. The efficacy and safety of HPV vaccines are well-documented, and Japan needs to revitalise its program. Due to resource constraints, only 20 %–30 % of LMICs have introduced HPV vaccination [43]. In Africa, school-based programs target 9–14-year-old girls [44,45], with 16 countries considering a single-dose regimen [46]; reducing doses and expanding coverage are key to preventing cervical cancer in LMICs [47,48].

The results of the Frontier analysis (Fig. 4) indicate that countries such as Palau, Zambia, Congo, Haiti, Guinea-Bissau, Mozambique, Eritrea, Zimbabwe, Lesotho, and Kiribati have considerable potential for reducing the burden of cervical cancer. A systematic review estimated that global coverage of cervical cancer screening (across 202 countries and territories) was 32 % in the past five years and 36 % over a lifetime [29]. Factors such as religious beliefs, limited partner involvement, low educational levels, lack of screening facilities, overburdened healthcare systems, inadequate equipment, and a shortage of medical personnel collectively hinder progress in eliminating cervical cancer in LMICs [49,50].

In addition, this study observed that the disease burden of cervical cancer decreases with increasing SDI (Fig. 3). Analysis of health disparities reveals that countries or regions with low SDIs experience higher incidence rates and DALYs, and this inequality is intensifying (Fig. 5), indicating a widening socioeconomic gap in access to effective prevention, screening, and treatment services. Epidemiological data show that cervical cancer is the leading cause of female cancer death in 42 resource-limited countries, but it ranks 19th in Finland [51], reflecting differential risk exposure and healthcare access. HPV vaccine coverage in underdeveloped regions is less than 3 % among females aged 10–20 years, compared to more than 33.6 % in developed regions[52]. Furthermore, only about 20 % of women in underdeveloped regions have undergone cervical cancer screening, whereas in developed regions, this figure exceeds 60 % [53]. This may be due to the allocation of funds for cervical cancer prevention and control in LMICs being less than 10 % of the estimated needs projected by the WHO for 2019–2030 [54].

ARIMA projections indicate high-SDI regions will not be able to reduce the age-standardised incidence rate of cervical cancer to below 4 per 100,000 within the next thirty years, presenting challenges for the WHO elimination targets. One contributing factor is the increasing proportion of cervical cancer cases among older women in high-SDI countries, where screening participation declines substantially beyond age 60. In fact, the risk of cervical cancer in 85-year-olds is dozens of times that in 70-year-olds, suggesting that elderly women need enhanced monitoring [55]. This demographic trend underscores the importance of age-tailored interventions, such as extending routine screening beyond current upper age limits, developing geriatric-friendly outreach programs, and integrating AI-based tools to improve detection among older populations. Without addressing this shift, elimination goals may remain out of reach despite overall system capacity. Nevertheless, the strategy has generated crucial momentum. Modeling suggests that HPV-vaccinated populations may eliminate cervical cancer as a public health issue by 2059–2102 [56]. Furthermore, adding two lifetime HPV screenings can reduce cervical cancer incidence from 2.1 to 0.7 cases per 100,000 women-years, increasing the overall reduction to 96.7 % and accelerating elimination by 11 to 31 years [56]. Australia’s free HPV vaccination program reduced infection rates among 18–24-year-old women from 22.7 % to 1.1 % by 2018, positioning it to become the first nation to eliminate cervical cancer by 2035 [57].

## Strengths and limitations

This study has several strengths. First, it is grounded in high-quality, standardised data from the GBD study, which offers comprehensive and comparable estimates across 204 countries and territories over a 32-year span. This global dataset ensures the robustness and representativeness of the analysis. Second, this study adopts a novel methodological approach by designating 2018—the launch year of the WHO’s global cervical cancer elimination strategy—as a pivotal timepoint to conduct segmented trend analyses (1990–2018 vs. 2019–2021). This design enables the early assessment of temporal shifts in disease burden following the global policy initiative, offering an innovative framework for evaluating real-world impacts of large-scale health strategies. Third, by applying the ARIMA model, this study forecasts future cervical cancer burden, providing forward-looking evidence that aligns with the WHO’s long-term elimination strategy and supports planning for future global interventions.

This study has limitations primarily concerning data quality and methodology. First, the data quality and reporting accuracy may vary significantly across different regions and countries, potentially introducing bias [58]. Second, due to structural limitations of the GBD database, this study was unable to analyze cervical cancer burden by histological subtypes (e.g., squamous cell carcinoma, adenocarcinoma, adenosquamous carcinoma) or adjust for important epidemiologic covariates, such as HIV prevalence, HPV vaccination coverage, and fertility rates. The GBD platform does not provide access to individual-level or country-specific raw input data, which precludes subtype-specific estimation and formal multivariable or multilevel modeling. In response to this limitation, we have outlined a preliminary research agenda, recommending the integration of cervical cancer burden data with histological subtypes and key epidemiologic covariates (such as HIV prevalence) to support more robust causal inference and inform targeted policy interventions. Third, although the ARIMA model has been widely used for epidemic forecasting [59], a key limitation is its inability to capture non-linear patterns [60]. The longer the forecasting period, the broader the 95 % CI becomes. External factors—including policy shifts, vaccine access improvements, screening optimization, and AI-telemedicine advances [61]—may exert nonlinear impacts on transmission trends, leading to inaccurate predictions [62]. Finally, a notable limitation of this study is the absence of data beyond 2021. The most recent GBD dataset publicly available extends only to that year. However, the years 2022 to 2025 represent a critical period, encompassing both the initial phase of the WHO’s cervical cancer elimination strategy and the ongoing impact of the COVID-19 pandemic. These two global events may have substantially disrupted HPV vaccination, screening, and treatment services worldwide. As such, the lack of data for 2022–2025 may limit the accuracy and completeness of our projections.

## Future directions

Moving forward, targeted interventions are imperative to mitigate the disparities identified in this study. High-burden, low-income settings—including Palau, Zambia, the Congo, Haiti, Guinea-Bissau, Mozambique, Eritrea, Zimbabwe, Lesotho, and Kiribati—require prioritized support to overcome structural barriers to elimination. Scaling HPV vaccination and cervical screening in these regions, along with phased, time-bound local targets, will be pivotal to narrowing global inequities. In parallel, further research should aim to link disease burden metrics with specific covariates such as histological subtypes and HIV prevalence. Such integrated datasets would support more robust causal inference using multilevel or regression-based approaches. Additionally, the timely release of updated global data—including GBD extensions beyond 2021—will be essential for reassessing cervical cancer trends in the post-pandemic context and informing responsive public health strategies. International collaboration should prioritize equitable resource allocation and cost-effective interventions—such as integrated vaccination and screening programs—thereby accelerating progress toward the WHO’s vision of eliminating cervical cancer as a public health threat.

## Conclusion

This study confirms the significant global importance and impact of the WHO strategy for the elimination of cervical cancer. Our analysis indicated that there has been a more pronounced decrease in both age-standardised incidence rates and DALYs since 2018. However, the situation remains challenging in countries and regions with lower SDI levels. Future implementation of the strategy should focus primarily on international cooperation and resource sharing, especially in developing more effective local health strategies and interventions in low-income countries.

## Data sharing statement

The data that support the findings of this study are openly available on the Global Burden of Disease (GBD) website. Researchers can access and download the GBD data utilized in these analyses by visiting the official GBD website at https://www.healthdata.org/.

## Fundings

Fujian Research and Training Grants for Young and Middle-aged Leaders in Healthcare; Joint Funds for the Innovation of Science and Technology, Fujian Province (Grant number: 2021Y9169, 2021Y9180, 2023Y9394); Special Health Subsidy of Fujian Provincial Finance Department (Grant number: 210020650502302); Fujian Provincial Natural Science Foundation of China (Grant number: 2023J011223, 2025J01203); Youth and Middle-aged Training of Fujian Provincial Health Technology Project (Grant number: 2021GGB014); The Startup Fund for Scientific Research, Fujian Medical University (Grant number: 2023QH2047); The National Key Clinical Specialty Construction Program of China (Gynecology). All funding parties did not have any role in the design of the study or in the explanation of the data.

## Compliance with ethics requirements

This article does not contain any studies with human participants or animals performed by any of the authors. The data used in this research are publicly available and de-identified, retrieved from the Global Burden of Disease (GBD) study database. Therefore, ethical approval and informed consent were not required.

## Declaration of competing interest

The authors declare that they have no known competing financial interests or personal relationships that could have appeared to influence the work reported in this paper.
