# Supplementary material for: Analyzing and forecasting global cervical cancer burden based on WHO’s elimination strategy: insights and projections from a 1990–2021 global burden of disease (GBD) study covering 204 countries and territories
Source: J Adv Res. 2025 Sep 25;84:895–907. doi: 10.1016/j.jare.2025.09.038 (PMC13227222; doi:10.1016/j.jare.2025.09.038)
Supplement: Supplementary Data 5 [file mmc5.zip › IHME_GBD_SDI_2021_INFO_SHEET_Y2024M05D16.pdf]

Institute for Health
Metrics and Evaluation

Data Release Information Sheet

Data Summary

Dataset name: Global Burden of Disease Study 2021 (GBD 2021) Socio-Demographic Index (SDI) 1950–2021

Date of release: May 16, 2024

Summary:

The Global Burden of Disease Study 2021 (GBD 2021), coordinated by the Institute for Health Metrics and Evaluation (IHME), estimated the burden of diseases, injuries, and risk factors for 204 countries and territories and selected subnational locations.

Developed by GBD researchers and used to help produce these estimates, the Socio-demographic Index (SDI) is a composite indicator of development status strongly correlated with health outcomes. It is the geometric mean of 0 to 1 indices of total fertility rate under the age of 25 (TFU25), mean education for those ages 15 and older (EDU15+), and lag distributed income (LDI) per capita. As a composite, a location with an SDI of 0 would have a theoretical minimum level of development relevant to health, while a location with an SDI of 1 would have a theoretical maximum level.

This dataset provides tables with SDI values for all estimated GBD 2021 locations for 1950–2021, as well as 2021 location quintile and reference SDI quintile values.

Acknowledgements

Contributing organizations:

- Global Burden of Disease Collaborative Network

Funders:

- Bill and Melinda Gates Foundation (BMGF)

Suggested Citation:

Global Burden of Disease Collaborative Network. Global Burden of Disease Study 2021 (GBD 2021) Socio-Demographic Index (SDI) 1950–2021. Seattle, United States of America: Institute for Health Metrics and Evaluation (IHME), 2024.

File Inventory

File Name	Description	Version Date
IHME_GBD_SDI_2021_SDI_1950_2021_Y2024M05D16.CSV	SDI Values: 1950-2021 [CSV]	May 16, 2024
IHME_GBD_SDI_2021_SDI_1950_2021_CODEBOOK_Y2024M05D16.CSV	Codebook - SDI Values 1950-2021 [CSV]	May 16, 2024
IHME_GBD_SDI_2021_SDI_1990_2021_Y2024M05D16.XLSX	SDI Values: 1990-2020 [XLSX]	May 16, 2024
IHME_GBD_SDI_2021_SDI_QUINTILES_Y2024M05D16.XLSX	SDI Quintile Values	May 16, 2024
IHME_GBD_SDI_2021_REF_QUINTILES_Y2024M05D16.XLSX	SDI Reference Quintile Values	May 16, 2024
IHME_GBD_SDI_2021_INFO_SHEET_Y2024M05D16.PDF	Data Release Information Sheet	May 16, 2024

Additional Information

Terms and Conditions

http://www.healthdata.org/about/terms-and-conditions

Contact Information

To request further information about this dataset, please contact IHME:

Address:
Institute for Health Metrics and Evaluation
Population Health Building/Hans Rosling Center
3980 15th Ave. NE, Seattle, WA 98195 USA
UW Campus Box #351615

Telephone: +1-206-897-2800

Fax: +1-206-897-2899

Email: data@healthdata.org

Website: www.healthdata.org

These files may be updated periodically, so we appreciate hearing feedback or additional information about how these data are being used.
